# Subtle Application of Electrical Field-Induced Lossy Mode Resonance to Enhance Performance of Optical Planar Waveguide Biosensor

**DOI:** 10.3390/bios11030086

**Published:** 2021-03-18

**Authors:** Yu-Cheng Lin, Liang-Yü Chen

**Affiliations:** 1 Electronic Engineering Department, Ming-Chung University, Taoyuan 33348, Taiwan; 2 Biotechnology Department, Ming-Chung University, Taoyuan 33348, Taiwan; loknath@mail.mcu.edu.tw

**Keywords:** lossy mode resonance, optical planar waveguide, electrical-field, biosensor, bovine serum albumin, sensitivity

## Abstract

Many studies concern the generation of lossy mode resonances (LMRs) using metallic oxide thin films that are deposited on optical fiber. However, the LMR-based optical fiber sensors are frangible, do not allow easy surface modification, and are not suited to mass production. This study proposes an electrical field-induced LMR-based biosensor with an optical planar waveguide to replace surface modification and allow the mass production of protein biosensors and accelerate the speed of the analyte to decrease the detection time. Experimentally, the biosensor is evaluated using charged serum albumin molecules and characterized in terms of the LMR wavelength shift using an externally applied voltage for different durations. The externally applied voltage generates a significant electric field, which drives the non-neutralized biomolecules and increases the LMR wavelength shift. Our experimental results demonstrate that there are two different mechanisms of adsorption of serum albumin molecules for short-term and long-term observations. These are used to calculate the sensitivity of the biosensor. This electrical field-induced method is highly significant for the development and fabrication of LMR-based biosensors.

## 1. Introduction

Thin films-coated optical sensors have been the subject of studies for decades and the development of biomaterials and immobilization methods for surfaces allow the development of new types of sensors. The first surface plasmon resonance (SPR) based sensor was proposed by Nylander et al. in 1982 [1]. It used a Kretschmann configuration, which consisted of an optical prism with a 56-nm-thick silver thin-film coating. SPR-based sensors are used for the inspection of biomaterials [2], chemical detection [3] and physical testing [4]. SPR occurs when the real part of the thin-film permittivity is negative and greater in magnitude than both its imaginary part and the real part of the permittivity of the sampling material. The optical spectrum of the SPR sensor is highly sensitive to the surrounding medium. If a layer that is sensitive to a biological or chemical analyte is immobilized on top of the metallic layer, a biosensor or a chemical sensor is achieved. However, the sensitivity of SPR sensors cannot be increased [5]. A finite one-dimensional photonic crystal (1DPC) structure for an electromagnetic waves propagating at the interface between a homogeneous medium, which are named Bloch surface waves (BSWs), have found a large number of applications [6,7,8] and were proposed as an alternative to SPR [9]. Contrary to SPPs, the BSWs can be excited by both s- and p-polarized waves by changing the geometry and materials of the photonic crystal. BSWs offer several possible advantages compared to SPP. Their dispersion can be designed at almost any wavelength by properly choosing the refractive index and thickness of the layers of the 1DPC. Owing to the dielectric materials of the photonic crystal, sensors based on BSWs are characterized by mechanical and chemical stability, thus permitting operation in aggressive environments [9]. However, 1DPC is a multi-layer stacked structure where a complex and precise coating process is required and that will increase the difficulty of sensor production. Lossy-mode resonances (LMRs) can be accomplished using a Kretschmann configuration. Dielectric waveguide modes and a lossy mode (a guided mode with a complex effective index) are combined to form a semiconductor-clad waveguide, as demonstrated by Marciniack in 1993 [10]. An optical fiber sensor that uses LMRs according to wavelength was demonstrated and the correlation between experiments and theory was verified in 2010 [11]. This lossy mode is not limited to a semiconductor cladding layer: it can be also observed for a dielectric cladding on a waveguide. LMRs occur when the real part of the cladding permittivity is positive and greater in magnitude than both its imaginary part and the real part permittivity of the sampling materials. The generation of LMRs using a metal oxide thin film that is deposited on optical fiber has been the subject of studies because resonance is generated for both TE (transverse electric) and TM (transverse magnetic) polarized light, so multiple resonances can be excited. The LMR wavelength can be tuned in the optical spectrum, so LMR-based optical fiber sensors can be used to detect the refractive index [12], humidity [13], chemicals [14], biological species [15], and pressure [16].

In an optical LMR sensor structure, the substrate is a prism, an optical fiber, a glass slab, or a waveguide. Optical fiber-based sensors are less costly and more portable than prism-based sensors and they allow remote detection and are resistant to electromagnetic interference. Most LMR sensors are constructed using optical fibers [17,18,19,20,21,22]. SPRs typically operate at angles between 40° and 70° [23], but LMRs typically operate at angles approaching 90°, which is adequate for optical fiber excitation. Therefore, LMRs cannot be excited using an equilateral prism or a right-angle prism if the light is not directed onto the lateral sides. Commercial prisms are not polished on the lateral sides, so are unsuited to LMR generation. Optical fiber-based LMR sensors are frangible and difficult to handle during cleaning procedures, coating processes and surface modification for the detection of certain analytes. A D-shaped optical fiber [22,24] or side-polishing to remove a portion of the cladding increases sensitivity. Side-polished silica fiber and manufacturing processes that involve cleaning and thin-film coating are not conducive to mass production so LMR sensors are limited in terms of applications. Recently, an optical planar waveguide is proposed as an alternative to optical fiber for LMR-based sensors because it is easier to handle, more robust, and lower cost [25]. It is regarded as opening up the path for the development of LMR-based chemical sensors, environmental sensors, and biosensors. Surface modification is another barrier to the development of sensors. In a chemical or biosensor, the sensing layer interacts with the target analyte after modification. The interaction between the modified surface and the analyte affects the optical properties of the sensing layer so it is detected by the sensor, which monitors the change in the refractive index to determine any change in the status of the analyte. A variation in the refractive index changes the wavelength at which resonance occurs. The LMR spectra are measured to monitor the analyte condition, which is the basic mechanism for LMR-based optical biosensors. Biosensors are used to detect specific biomolecules but a series of tedious probe immobilization or surface modification processes must be completed in advance. This procedure is quite time-consuming.

Biological detection relies on diffusion and random collisions to achieve biological or chemical reactions so it often takes hours or days to complete a detection if there are no external forces. The external force that is required to accelerate the aggregation of the molecules to be detected must be applied to increase sensitivity and the sensing speed. Many biomolecules are composed of protein structures and are electrically neutral so protein molecules must be charged if an external electric field is used to accelerate the movement of the protein. Generally, proteins have a lower isoelectric point (bovine serum albumin (BSA) with an isoelectric point of 4.7) and must be dissolved to achieve negative charge in a buffer with a higher pH value (phosphate-buffered saline (PBS) with pH 6.9), according to the characteristics of protein isoelectric point. The protein is negatively charged so if a negative voltage is applied to the upper electrode of the sensor and a positive voltage is applied to the bottom electrode, the negatively charged protein is driven by the electric field to the bottom positive electrode and electrophoresis occurs. The protein molecules are adsorbed onto the surface of the lower electrode and the LMR wavelength shifts so the analyte protein is detected without the need for surface modification.

This study addresses problems that are inherent to LMR-based optical fiber biosensors. Surface modification is a lengthy process that prevents the mass production of biosensors and a simple and robust sensor structure must be used to solve the problem of fragile optical fiber and the difficulty of surface modification. An external electric field is necessary to accelerate the speed of the analyte and decrease the detection time. This study demonstrates an electrical field-induced lossy mode resonance-based optical planar waveguide (EF-LMROPW) biosensor that uses an external electric field to drive charged protein molecules and uses the sensing principle of LMR. The biosensor consists of a self-lighting alignment mechanism in a complete biosensing platform. Optical planar waveguides have a planar geometry to guide light and are often fabricated as a thin transparent film. Waveguides are made of dielectric materials and the guiding layer has a greater refractive index than those of the two bounding media, to allow total internal reflection for confined propagation. For an EF-LMROPW sensor, the planar waveguide layer is a glass slab, one bounding medium is air and the other is the indium tin oxide (ITO) thin film that is attached using an analyte solution. The ITO material is stable for coating and is widely applied in the fabrication of transparent conductive thin films. The proposed sensor structure is fabricated on an ITO conductive glass plate. The ITO film serves as the LMR dielectric material and also as the bottom electrode for the EF-LMROPW biosensor. The surface of the upper substrate is pasted with a metal layer as the upper electrode and the upper and lower substrates are separated by insulating spacers. When an external voltage is applied between the two electrodes, an electric field drives the charged protein molecules. Experimentally, the biosensors are evaluated using BSA solution and characterized in terms of the dynamic LMR wavelength shift for a specific externally applied voltage. To the authors’ best knowledge, no study has been published that uses an electrical field for an LMROPW to produce a biosensor.

## 2. Materials and Methods

### 2.1. Materials

LMRs are generated by guiding modes that are propagated in a coating that is deposited on a soda-lime glass slab waveguide. Soda-lime glass has very high transparency for wavelengths from 400 to 800 nm [26], which allows monitoring using less expensive optical light sources and a spectrometer. The sputtering process used square glass with a thickness of 0.4 mm and a length and width of 100 mm. BSA and PBS were purchased from Uni-Onward Corp. All of the chemicals were analytical grade and used without purification. The aqueous solutions were prepared using ultrapure water (>18.2 MΩ-cm) and a Milli-Q system (Burlington, MA, USA). Some BSA was stirred and dissolved in PBS for 30 min at room temperature using an electromagnetic stirrer to prepare a 1% BSA solution. When a hydrogen atom loses an electron, it becomes a positively charged proton. This proton can attach to a group with a high electron density (such as an amino group) and this group has an extra positive charge. The proton can also easily escape from specific groups (such as an acid group), and the group becomes negatively charged. Protein molecules have both positive and negative charges and the net value of these charges is the static charge of the protein. This static charge depends on the pH of the environment. At a specific pH value, the electrostatic charge on the surface of the protein is 0. This pH value is defined as the PI of the protein. The PI value is an important indicator of the charged properties of proteins and the environment affects these properties. If the pH value of the environment is greater than the PI, the net charge is negative; otherwise, it is positive. Proteins in cells have the PI between 4 and 6, so if they are in a neutral environment, they are negatively charged. The PI for BSA is 4.7 and when it is dissolved in PBS buffer, the pH is 6.9. The PBS solution dissociates some potassium ions (K^+^) and phosphate ions (PO_4_^3−^) and an environment of PBS causes the BSA to be negatively charged.

### 2.2. ITO Thin-Film Coating

Before sputtering, all of the soda-lime glass squared sheets with a thickness 0.4 mm and a side length of 100 mm (Liefco Optical Inc., Taichung, Taiwan) were pre-cleaned with acetone using purity wipes. The glass sheets were then placed in an aqueous solution of 10% NaOH at 55 °C for 3 min, rinsed in deionized water for about 1 min and then checked to ensure that water sheeted off the glass surface immediately. The glass sheets were wiped dry to prevent water spots and were used as the substrate for sputter coating. IZOVAC Co. equipment used a partial pressure of argon of 9 × 10^−2^ mbar and an intensity of 150 mA. The resulting station used a gas system based on 2 MFC (Ar and O_2_). Each magnetron had a 3-zone gas distribution system with a manual needle valve for uniform adjustment. The magnetrons were powered by 10 kW DC power supplies. The base vacuum before the process was 5 × 10^−3^ Pa. The operating gases were argon and oxygen of 99.99% purity. The ITO target of 99.99% purity was purchased from Solar Applied Materials Technology Co. After sputtering, the ITO glass sheets were cut into small pieces of 30 mm squares as LMR components. The ITO was about 100 nm thick, which was measured using an optical interferometer.

### 2.3. EF-LMR Biosensor Assembly

**Figure 1:**
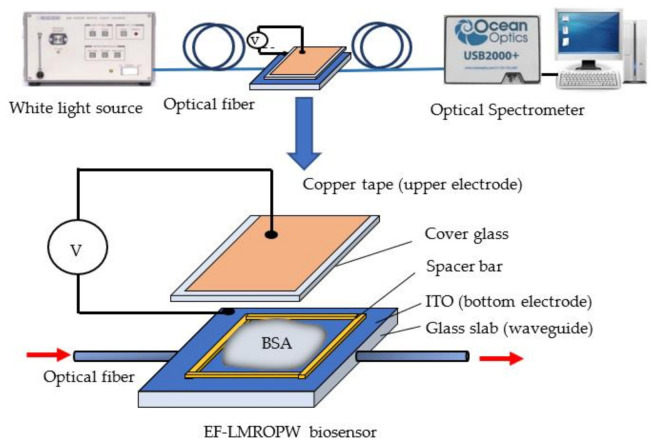
A schematic diagram of the experimental set-up and a photograph of the electrical field-induced lossy mode resonance-based optical planar waveguide (EF-LMROPW) sensor with the optical alignment platform.

To generate an electric field to drive charged protein molecules, two (upper and bottom) planar electrodes were assembled and insulated. The ITO film material is conductive so it can be coated on a planar glass sheet as the bottom electrode. The upper electrode used a cover glass with a thickness of 0.15 mm on which a square of copper foil tape with a thickness of 50 μm and sides of 22 mm were attached. A strong electric field requires a small gap between the two electrodes. A PET thin film with a thickness of 0.15 mm was applied around the glass surface as a spacer bar to separate the upper and lower electrodes. The gap between the two electrodes was 0.3 mm: the thickness of the PET (0.15 mm) plus the thickness of the cover glass (0.15 mm). The upper electrode was connected to the power supply using copper foil tape and the bottom electrode was connected to the power supply using silver glue on the ITO surface. An EF-LMROPW sensor that produces an electric field was realized. The structural exploded view of the EF-LMROPW, showing the hollow cell that is formed between the two electrodes, is shown in Figure 1.

### 2.4. LMR Experiment Setup

A typical optical transmission measurement setup was used to measure the spectra that are transmitted through the sensor. Halogen white light (4303B, ANDO Co. Ltd., Kanagawa, Japan) with a 400~1800 nm wavelength was used as the input source and an optical spectrometer (USB 2000, Ocean Optics^®^, Dunedin, FL, USA) was the receiver to measure the spectrum of the EF-LMROPW sensor. A schematic diagram of the experimental setup is shown in Figure 1. For ease of self-alignment, the bulk platform was designed to fit all components and the optical axis was along the center of the optical planar waveguide (OPW). An optical fiber patch cord (M74L01), which has a core/cladding diameter of 400/425 μm and NA 0.39 purchased from THORLABS, guiding light from the input light source was inserted into the hole at the left side of the platform with an FC connector. Another optical fiber patch cord (QP600-1-UV-VIS), which has a core diameter of 600 μm purchased from Ocean Optics Co., was inserted into the right side of the platform and connected to the spectrometer with an SMA connector. The platform aligns the optical fiber to the OPW and then the OPW to the optical fiber so the dimension was designed in advance for fine fitting. In terms of lateral or vertical alignment, since the machining error is less than 10 μm, which is also much less than the thickness of the waveguide (0.4 mm) and the diameter of optical fiber (400 μm), misalignment could be neglected.

A charged BSA solution was dropped onto ITO glass and the upper plate electrode was covered. The voltage was provided by a DC power supply (LPS-305, American Reliance Co., Los Angeles, CA, USA). The bottom electrode was connected to the positive voltage and the upper electrode was connected to the negative voltage. The power supply voltage was increased from 0 to 180 V in steps of 30 V. The wavelength-dependent transmission of the sensor in various analytes was normalized using the spectrum for the bare sensor surrounded by air. The EF-LMROPW sensor was washed with DI water after each measurement to avoid interference from leftover BSA molecules on the ITO surface.

## 3. Results and Discussions

The LMR wavelength is observed in the transmission spectrum. The LMR wavelength interrogation method correlates to determine the dip in the transmission spectrum for EF-LMROPW sensors. The transmission is defined as the ratio of the transmitted light intensity for a specific analyte to the reference incident light intensity in the air. Charged BSA molecules are driven by an external electric field so BSA moves to the bottom electrode to maintain a dynamic balance; each measurement was repeated 10 times. The measurement uncertainty is used to calculate the mean value and the standard deviation. The error tolerance is twice the standard deviation, as shown in all figures. The measured results have a 95% confidence level to demonstrate the dynamic balance of the status of the biomolecules.

### 3.1. Observations with Short-Term Applied Voltage

**Figure 2:**
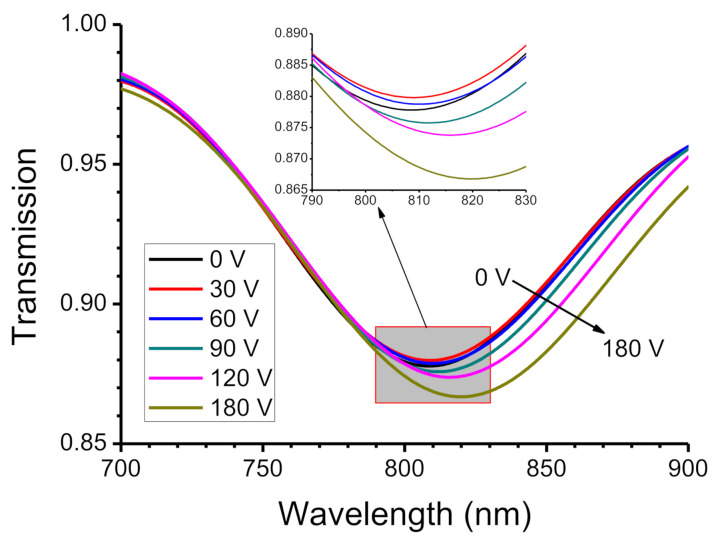
The transmission spectra for 1% bovine serum albumin (BSA) solution for external voltages from 0 to 180 V.

**Figure 3:**
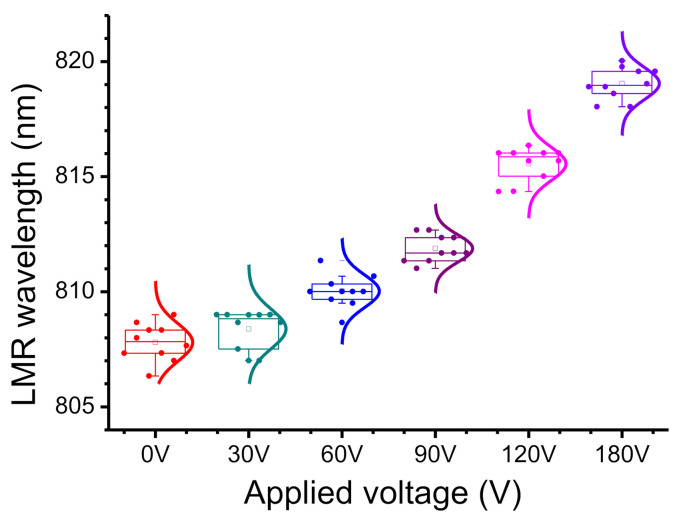
The distribution of lossy mode resonances (LMR) wavelengths for 1% BSA for applied voltages from 0 to 180 V.

To determine the effect of the electric field on the movement and adsorption of BSA on the electrode, the 1% BSA solution was dropped into the hollow structure between the upper and lower electrodes. The applied voltage was varied from 0 to 180 V in steps of 30 V, which is equivalent to increasing the electric field intensity from 0 V/cm to 6 kV/cm in steps of 1 kV/cm. A delay of 10 s allowed movement of BSA molecules and then the spectra were recorded 10 times to calculate the mean and standard deviation for the LMR wavelength. The measured transmission spectra are shown in Figure 2. The point at which the curve dips is the LMR wavelength. The initial resonance wavelength is 808.6 nm, without an applied voltage (0 V), and there is a red-shift to 819.0 nm when the applied voltage is 180 V. The total shift in the LMR wavelength is 10.4 nm. The transmission also becomes lower and obvious, which decreases from 0.88 (0 V) to 0.86 (180 V), according to the increase in applied voltages. Figure 3 shows the distribution of the LMR wavelength for the six applied voltages. The statistical error tolerance (that is, twice the standard deviation) is between 0.59 and 0.86 nm. Compared with the value for V = 0, the LMR wavelength for an applied voltage of 30 V increases by only 0.5 nm, which is less than the error tolerance. It is because the electric field of 1 kV/cm that is generated by a voltage of 30 V is too weak to allow the charged BSA molecules to move to the ITO surface in a short time. The electric field that is generated by an applied voltage of more than 60 V is strong enough to move the BSA molecules to the ITO surface so the LMR wavelength shifts significantly.

**Figure 4:**
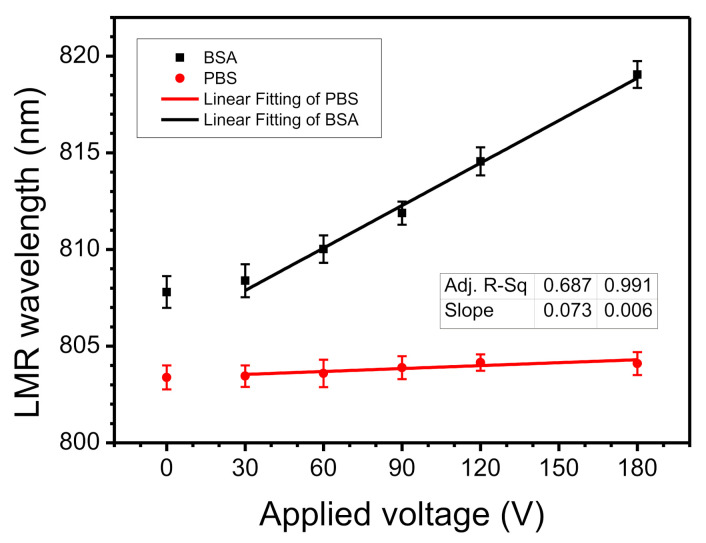
Variation in LMR wavelength shift for 1% BSA and phosphate-buffered saline (PBS) solution for 100 s using applied voltages from 0 to 180 V.

To determine whether the shift in the LMR wavelength is due to the charged BSA molecules being driven by the electric field and becoming adsorbed onto the bottom electrode, the same measurement was performed using pure PBS solution (without BSA). The experimental results are shown in Figure 4. As the voltage increases, the LMR wavelength for PBS does not vary significantly but there is a greater LMR wavelength shift for 1% BSA. The maximum change (*∆λ*) is 0.7 and 11.3 nm, respectively. This slight increase in PBS aqueous solution may be caused by phosphate ions, which approximate a linear approach. The wavelength shifts when a voltage is applied to BSA can be fitted by an exponential function (R^2^ 0.990), as shown in Figure 4. The wavelength was measured for a total of five voltages and the entire measurement time was about 100 s. The wavelength shift of 11.3 nm at 180 V corresponds to a voltage sensitivity (wavelength shift per unit voltage) is 0.062 nm/V in the short-term. These results show that when an electric field is applied, the protein is adsorbed onto the bottom substrate and the applied voltage has an exponential relationship with the shift in the LMR wavelength.

### 3.2. Observations with Long-Term Applied Voltage

**Figure 5:**
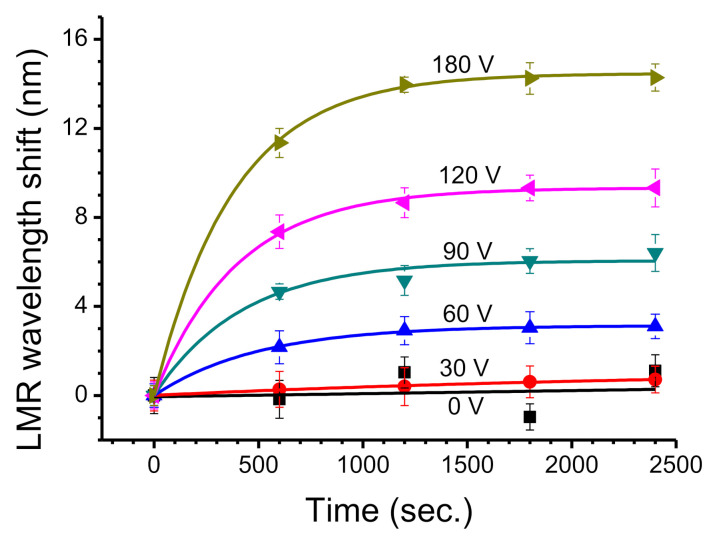
LMR wavelength shifts for 1% BSA for an external voltage from 0 to 180 V for 100 min.

Negatively charged BSA molecules are driven by an external electric field. Different electric field intensities exert a different force on BSA molecules and they move at different speeds, so they require different periods of time to reach the bottom positive electrode. To determine the LMR wavelength shift due to BSA molecules for the long-term, the shift for six applied voltages from 0 to 180 V was measured. The measurements were repeated every 600 s for ten times. Each voltage was applied for 6000 s. After more than 2400 s, the resonance wavelength for LMR was very stable. The plots only show the state of the LMR wavelength shift in the first 2400 s to determine the transient state for the previous period, as shown in Figure 5. When the externally applied voltage is 0 V, the LMR wavelength does not increase. For an external voltage of 30 V, the wavelength shifts increase by about 0.72 nm. For an external voltage of 60 V, the LMR wavelength increases by nearly 3.04 nm after 1800 s.

An applied voltage of 120 and 180 V induces a stronger electric field, which causes a significant LMR wavelength shift of 7.36 and 11.34 nm in the 600th second, respectively. After 1800 s, the respective LMR wavelength shifts for 120 and 180 V are 9.32 and 14.24 nm, respectively, and remain stable. For these measurements, the error tolerance is between 0.34 and 0.85 nm. The period of time that is required to reach a stable state shows that a stronger electric field quickly drives the charged molecules of BSA. A stronger electric field causes the bottom electrode to adsorb and stack more protein molecules so there is a change in the surface state of the ITO and the LMR wavelength shift increases. The entire shifts in LMR wavelength satisfy exponential fitting with R^2^ values between 0.966 and 0.999.

### 3.3. Sensitivity and Molecular Kinetics

Negatively charged BSA molecules are driven by an externally applied voltage (*Va*) so they are attracted to the positively charged ITO surface. The absorbed BSA, *Q(t)*, gradually accumulates on the interface. The saturated adsorption capacity for BSA is *Qmax* for each *Va* so the molecular coverage on the interface is defined as Equation (1), which is a function of time:

(1) $$\eta \left(t\right)=\frac{Q\left(t\right)}{Q_{max}}$$

The quantity that is adsorbed onto the ITO electrode changes the propagating loss mode and results in the shift in the LMR wavelength (*Δλ*). This tiny molecular cumulant *Q* and the wavelength shift are expressed in a linear relationship as Equation (2), where *μ* is the linear relationship between *Q(t)* and *Δλ(t)*:

(2) $$Q\left(t\right)=\mu \Delta \lambda \left(t\right)$$

The maximum value for the LMR wavelength shift is *Δλ_max_* so the adsorption coverage for BSA is also defined by the relationship between the LMR wavelength shift as Equation (3):

(3) $$\eta \left(t\right)=\frac{\Delta \lambda \left(t\right)}{\Delta \lambda_{max}}$$

The sensitivity of the EF-LMR sensor is defined as the LMR wavelength shift *Δλ* due to an applied unit voltage (*ΔV*). The sensitivity is affected by the equivalent driving voltage ($V_{eff}$) and the duration of the applied voltage, which is a function of the applied voltage. Therefore, sensitivity has a short-term (α_short_) and long-term (α_long_) component, as defined by Equation (4), where the equivalent voltage $V_{eff}$ is the effective potential that is added to the BSA molecules:

(4) $$\alpha {\left(V_{eff}\right)}_{short,long}=\frac{\Delta \lambda }{\Delta V}=\frac{Q/\mu }{\Delta V}$$

In the initial stage when BSA is not adsorbed onto the ITO surface, $V_{eff}$ is equal to the externally applied voltage. As the negatively charged molecules begin to adhere to the surface of the ITO, a negatively charged built-in potential ($V_{build-in}$) is created to offset the original applied voltage (*V_a_*). Therefore, the equivalent voltage is expressed as Equation (5):

(5) $$V_{eff}=V_{a}-V_{build-in}$$

The built-in potential gradually increases with the duration of the external voltage so the sensitivity is different for long-term and short-term application of an external voltage. A voltage duration of 100 s is defined as short-term and a duration of 2400 s is long-term. The short-time sensitivity, $\alpha_{short}$, is calculated using the data in Figure 4 to relate the wavelength shift *Δλ_short_* for each increase of *ΔV*. The results are listed in Table 1. As the applied voltage increases from 30 to 180 V, *α*_short_ increases from 0.019 to 0.062 nm/V. Therefore, at high voltage, the charged molecules are driven by a stronger electric field and are directly adsorbed onto the ITO surface in large quantities. For short-term duration, the coverage is low and there is no saturation so that the built-in voltage is negligible.

In Table 1, *Δλ_max_* is the maximum value for the wavelength shift and *k* is the kinetic parameter, which is related to the applied voltage and time. Using Equation (6), the *k* value is calculated:

(6) $$k=-\frac{ln\left(1-r\right)}{t}$$

where $r=\frac{\Delta \lambda_{long}\left(t\right)}{\Delta \lambda_{Max}}\;$ is the ratio of the LMR wavelength shift. The long-term experiment shows that Δλ_long_(t) increases with time for a fixed voltage, as shown in Figure 5. For long-term observation at 30 V in 6000 s, the maximum LMR wavelength shift (*Δλ_max_*) is only 1.18 nm. When the applied voltage is 180 V, the maximum wavelength shift increases to 14.80 nm. The long-term sensitivity ($\alpha_{long}$) is calculated using the LMR wavelength shift and the results are shown in Table 1. The long-term sensitivity is greater than the short-term sensitivity. If the applied voltage is less than 60 V, the former is about twice as large as the latter. At more than 90 V, the former is only about 1.5 times the latter. This multiple gradually decreases as the applied voltage increases. There is more molecular stacking if there is long-term adsorption of BSA on the electrode surface under a high voltage so more negative charge accumulates. This creates a higher built-in potential, which reduces the effective voltage and affects the long-term sensitivity. According to the data in Figure 5, the LMR wavelength shift could be converted into a ratio r, which is used to calculate the apparent *k* value by Equation (6). This shows the kinetic parameter for each applied voltage, as listed in Table 1. For the long-term experiment, the main wavelength shift occurs in the first 600 s. This includes the short-term adsorption mechanism in the first 100 s and then gradually enters the process of long-term molecular adsorption. The *k* value slowly decreases as time increases.

In terms of molecular kinetics, there are two mechanisms for the adsorbed molecules by the spatial distributions over time [27]. For the first 100 s, the molecules near the bottom electrode are adsorbed immediately when a voltage is applied. At the initial stage, fewer molecules are stacked and the *k* value is large. As the time exceeds 100 s, the charged molecules at a distance are pushed towards the bottom electrode. The electrostatic force that is generated by the electric field is similar to electrophoresis. When the molecules reach the bottom electrode, a molecular stack is formed so the built-in potential increases and there is a slow LMR wavelength shift and a smaller *k* value.

### 3.4. Molecular Desorption with No Applied Voltage

**Figure 6:**
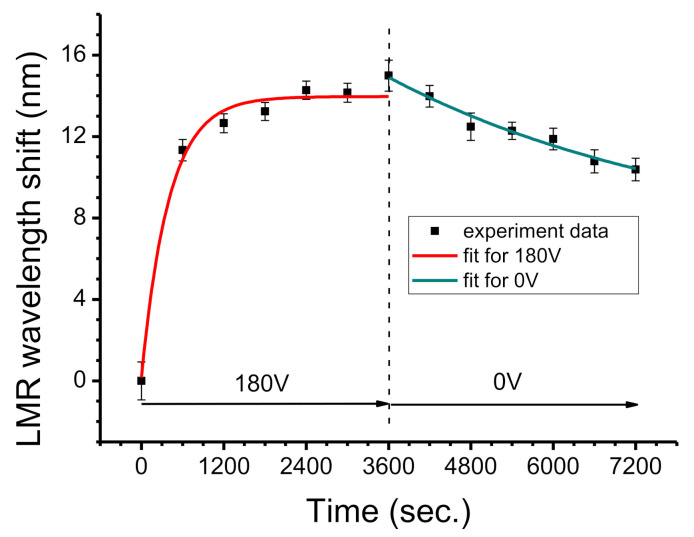
LMR wavelength shift of 1% BSA for an applied voltage 180 V for 3600 s and with no applied voltage for another 3600 s.

The charged BSA molecules were driven by an external voltage of 180 V for 3600 s, and then the external voltage was turned off (*V* = 0) for another 3600 s. The experimental results are shown in Figure 6. In the first 3600 s, the charged BSA is adsorbed by the bottom electrode and the LMR wavelength increases by 15.0 nm. When the power is turned off, the wavelength decreases slightly. After 3600 s, it decreases by 4.6 nm because charged BSA molecules are no longer driven by an external electric field. These negatively charged BSA molecules repel each other and diffusion causes the gradual desorption of BSA from the bottom electrode and a return to the PBS solution. Finally, it reaches a thermodynamic equilibrium. The adsorption mechanism under an applied voltage (180 V) is different from the desorption mechanism without an applied voltage (0 V) so two exponential fitting curves are plotted individually for these two sets of data where the *R^2^* values are 0.9545 and 0.9588, respectively.

The concentration of BSA is very critical for biosensing. Compared with other biological sensing technologies, the external electric field of the EF-LMROPW sensor can effectively enhance the non-specific adsorption of any charged molecules. In medical clinics, BSA usually acts as a carrier for drugs and delivers them to animal organs. Therefore, a BSA molecule usually works as a model biomolecule. Regarding the issue of BSA concentration, many reports were presented. For example, in humans, the concentration of the most abundant serum protein, albumin, is 50 mg/mL (5%) and comprises about half of the total protein mass [28]; human serum albumin is synthesized in and secreted from the liver. It is the most abundant plasma protein and the concentration is typically 35 to 50 g/L (3.5 to 5.0%) [29]. In our experimental measurement for 1% BSA concentration, the resolution of the spectrum is 0.346 nm, converted into the amount of protein adsorption, the concentration resolution can reach 0.02% (200 ppm). The EF-LMROPW biosensor sensitivity is sufficient for the detection of the clinically relevant concentration of the selected proteins. In the future, the sensing and analysis methods of adsorption for specific protein molecules are the goal of our development for this EF-LMROPW biosensor.

## 4. Conclusions

An EF-LMROPW biosensor is experimentally demonstrated for the first implementation with an ITO-coated optical planar waveguide and a pair of electrodes for applying external voltage. It features free surface modification, high mechanical stability and a simple fabrication process so the EF-LMROPW biosensor under the driving of an externally applied voltage is a voltage-controllable device that is suited to mass production. An adjustable applied voltage and charged analyte allow the detection of biomolecules without a need for complex surface modification. Our results show that the novel component with external electric fields can greatly enhance the sensitivity and performance of biosensors.

## Figures and Tables

**Table 1:** Kinetics of short-term and long-term for applied voltages.

Va (Volt.)	Short-Term		Long-Term			
	*Δλ_short_* (nm)	α_short_	*Δλ_max_* (nm)	α_long_	*k*	*R* ^2^
30	0.585	0.019	1.177	0.039	0.0005	0.980
60	2.224	0.037	3.862	0.064	0.0014	0.933
90	4.080	0.045	6.862	0.076	0.0019	0.970
120	6.756	0.056	9.788	0.081	0.0023	0.993
180	11.247	0.062	14.796	0.082	0.0024	0.997

## Data Availability

Data is contained within the article.
